# The first nationwide survey of MD-PhDs in the social sciences and humanities: training patterns and career choices

**DOI:** 10.1186/s12909-017-0896-1

**Published:** 2017-03-21

**Authors:** Seth M. Holmes, Jennifer Karlin, Scott D. Stonington, Diane L. Gottheil

**Affiliations:** 1 Public Health and Medical Anthropology, University of California Berkeley, 50 University Hall, MC 7360, Berkeley, CA 94720 USA; 2 Anthropology, History and Social Medicine, School of Medicine, University of California San Francisco, 50 University Hall, MC 7360, Berkeley, CA 94720 USA; 3 Department of Medicine, Alameda County Medical Center, 50 University Hall, MC 7360, Berkeley, CA 94720 USA; 4 Family and Community Medicine, School of Medicine, University of California San Francisco, San Francisco, USA; 5 Department of Anthropology and Department of Medicine, School of Medicine, University of Michigan, Michigan, USA; 6 Medical Scholars Program, University of Illinois College of Medicine at Urbana-Champaign, Illinois, USA

**Keywords:** MD-PhD training, Physician-investigators, Medical education, Social science, Humanities

## Abstract

**Background:**

While several articles on MD-PhD trainees in the basic sciences have been published in the past several years, very little research exists on physician-investigators in the social sciences and humanities. However, the numbers of MD-PhDs training in these fields and the number of programs offering training in these fields are increasing, particularly within the US. In addition, accountability for the public funding for MD-PhD programs requires knowledge about this growing population of trainees and their career trajectories.

The aim of this paper is to describe the first cohorts of MD-PhDs in the social sciences and humanities, to characterize their training and career paths, and to better understand their experiences of training and subsequent research and practice.

**Methods:**

This paper utilizes a multi-pronged recruitment method and novel survey instrument to examine an understudied population of MD-PhD trainees in the social sciences and humanities, many of whom completed both degrees without formal programmatic support. The survey instrument was designed to collect demographic, training and career trajectory data, as well as experiences of and perspectives on training and career. It describes their routes to professional development, characterizes obstacles to and predictors of success, and explores career trends.

**Results:**

The average length of time to complete both degrees was 9 years. The vast majority (90%) completed a clinical residency, almost all (98%) were engaged in research, the vast majority (88%) were employed in academic institutions, and several others (9%) held leadership positions in national and international health organizations. Very few (4%) went into private practice. The survey responses supply recommendations for supporting current trainees as well as areas for future research.

**Conclusions:**

In general, MD-PhDs in the social sciences and humanities have careers that fit the goals of agencies providing public funding for training physician-investigators: they are involved in mutually-informative medical research, clinical practice, and teaching – working to improve our responses to the social, cultural, and political determinants of health and health care. These findings provide strong evidence for continued and improved funding and programmatic support for MD-PhD trainees in the social sciences and humanities.

**Electronic supplementary material:**

The online version of this article (doi:10.1186/s12909-017-0896-1) contains supplementary material, which is available to authorized users.

## Background

In the 1950s and 1960s, combined Doctor of Medicine and Doctor of Philosophy (MD-PhD) training programs emerged in the United States with the goal of producing physician-investigators whose research was informed both by their scholarly discipline and their clinical activities, responding in novel ways to questions of critical medical importance [1]. In 1964, the United States National Institutes of Health (NIH) created the Medical Scientist Training Program (MSTP), one prominent face of the national agenda to train physician-investigators. Given the significant public investment through the MSTP, it is important to determine whether or not these training programs are meeting their objectives: namely producing the so-called “triple threat” researcher, clinician, and educator [2]. To answer this question, several recent studies have tracked the career outcomes of jointly trained MD-PhDs [1, 3–8]. While the MSTP oversees the training of physician-investigators in the basic biological, chemical and physical sciences and also the “social and behavioral sciences, economics, epidemiology, public health, bioengineering, biostatistics, and bioethics” [9], most of the studies in the literature to date have focused almost exclusively on the training and career outcomes of MD-PhDs in formal dual training programs in medicine and the biological, chemical and physical basic sciences. There has been very little research investigating the training and career outcomes of the growing number of MD-PhD trainees in the social sciences and humanities (SSH).

Formal MD-PhD training in SSH began with 3 programs in the 1970s and expanded, especially over the past 10 years, to 17 programs currently in the U.S. and Canada [10]. Over half (9 of 17) of these programs receive NIH MSTP funding while the other programs receive NIH funding through other mechanisms [10]. However, many MD-PhDs in SSH train outside of formal programs. MD-PhD physician-investigators in SSH receive degrees in diverse disciplines such as history, anthropology, philosophy, sociology, and economics. These physician-investigators are trained to use their disciplinary methods to analyze the ways in which disease, health and healthcare are influenced by social, cultural, political and economic forces. The increasing number of people pursuing this career path aligns with growing recognition that health and disease are determined by significant social and economic inequalities nationally and globally [11–16]. Indeed, several national institutions have acknowledged the need for physicians with a diverse array of analytic methods and novel tools to evaluate complex health systems, policies, and disparities [17–21]. The federally funded Clinical and Translational Science Awards (CTSA) aim to move biomedical research from “bench to bedside to curbside” and physician-investigators in SSH may prove critical to both of these translational steps to improve health outcomes [22–24]. Furthermore, current requirements for standardized testing and entering medical school now include the social sciences and humanities [25]. Possibly also spurred by several prominent MD-PhDs in SSH including Jim Kim (President of the World Bank), Paul Farmer (Co-Founder of Partners in Health), and Camara Jones (Medical Officer at the Centers for Disease Control and Prevention and President of the American Public Health Association), the interest in MD-PhD training in SSH is growing dramatically.

While physician-investigators in SSH are trained to answer critical health and healthcare problems in the contemporary world, and significant NIH MSTP monies are committed to such training, there has been little research on this growing cadre of physician-investigators, their careers, and their contributions to scholarship and society. In this study, we focus on the first cohorts of MD-PhD graduates in SSH inside and outside formal training programs in order to characterize and describe this population, provide insights about their educational trajectories and career outcomes, and assess the relationship between their career outcomes and the overall goals of public funding for MD-PhD training. In addition, this research into the training and careers of the first cohorts of MD-PhDs in SSH is necessary context and baseline to understand new trends in these pathways, including the dramatic increase in number of applicants for and trainees in these fields and what this signals for the future.

## Methods

- Design
  This project was designed to describe and analyze the population of MD-PhD graduates in SSH who trained in and outside formal MD-PhD programs, as well as explore their educational trajectories and career outcomes, and elicit their experiences of their training and careers.
- Measures
- The questionnaire (see Additional file 1) contained 14 questions that included demographic information, quantitative Likert-scale questions regarding satisfaction with training, levels of institutional support, degree of integration of dual careers, sequence of doctoral studies, residency specialty, and percent time devoted to clinical, research, teaching and other activities. In addition, it contained six open-ended qualitative questions concerning training experiences, obstacles, supports, and recommendations for the future. Due to the novelty of the study population, validated research tools were not available. The questionnaire was developed specifically for MD-PhDs in SSH. The questionnaire was developed by MD-PhDs in SSH and mixed method medical education scholars and then piloted among our study population to ensure clarity and consistency of understanding of items prior to recruitment and questionnaire administration.
- Recruitment
  In the absence of a preexisting, formal database of MD-PhDs, we employed a multi-pronged recruitment method to access physician-investigators who trained inside and outside formal programs. The recruitment method included three components: (a) utilizing formal MD-PhD training program records, (b) conducting targeted internet searches, and (c) soliciting participation through national conferences for MD-PhDs in SSH. MD-PhD training program records and contact information were requested from directors of such programs based in the US. Internet searches to identify MD-PhDs were conducted using variations of “MD-PhD” along with terms denoting diverse SSH disciplines. Finally, participants were identified via two national conferences for MD/PhDs in SSH. The latter two methods of recruitment were especially important to capture individuals who did not train within formal programs and therefore would not otherwise be represented in MD-PhD training program records. During recruitment, it was explained to participants that data may be used for publication in book or article form.
- Participants
  This work sought to understand the experiences and career trajectories of the first cohorts of MD-PhDs in SSH who trained as formal programs were first being established in the US. To this end, we focused our research on physician-investigators who finished their training by 2000 whether inside or outside a formal MD-PhD program. This enabled the research team to capture a baseline sample of the earliest cohorts of trainees in these fields while reducing generational bias. SSH fields were defined as academic disciplines, excluding explicitly professional fields such as public health and management; we included public policy and health services research as academic disciplines in this study as they highlight theoretical or generalizing foci. In the context of the US, the MD is the degree completed to become a physician. The MD is completed after a four-year, undergraduate university bachelor’s degree. The MD is a four-year program involving in-depth medical science courses as well as inpatient and outpatient clinical rotations. In the US, stand-alone PhD programs in SSH may last between 4 and 10 or more years.
- Procedure
  Questionnaires were delivered to participants, filled out by participants, and returned to the research team to be analyzed.
- Analysis
  Analyses focused on descriptive statistics, including the number and proportion of sampled individuals completing specific training paths, career paths, disciplines, and clinical specialties. Descriptive means and proportions were generated. We tested differences in these components by whether the respondent was optimistic about the ability of people to combine significant professional responsibilities in medicine and SSH. Participant responses to open-ended questions about optimism were dichotomized as ‘optimistic’ or ‘not optimistic’, and differences in continuous outcomes (e.g., year of graduation and time spent in school) were tested with t-tests; differences in nominal outcomes (e.g., clinical components of work post graduation) were tested with odds ratios and 95% confidence intervals. Statistical significance was set at *p* < 0.05. Open-ended, qualitative responses were coded utilizing grounded theory [26]. Grounded theory involves a process of coding data utilizing themes that emerge deductively from the data through cycles of increasing precision. Data with a single code were compiled and analyzed for their characteristics and meanings. Qualitative data analysis was performed with cross-checking by five scholars in the fields of medical education, medical humanities, and medical social sciences. Consensus regarding analysis was achieved through discussion of themes arrived at separately by each of the individuals involved. Qualitative responses were bundled into broad themes to facilitate presentation. Means and frequencies of these broad themes were generated. Forms of presentation of these data are consistent with common practice within qualitative health research and motivated by a desire to maintain fidelity to the data and clarity to the reader [27].

## Results

### Participation rate and general characteristics of participants

**Fig. 1:**
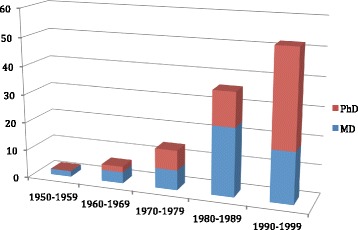
Absolute number of MD and PhD graduations by decade

A total of 63 physician-investigators were identified and contacted, of whom 55 completed surveys for a response rate of 87%. Although there is no national database to determine the size of this population, informal estimates of the total number of physician-investigators in SSH nationally range from 100 to 150 [28]. The 55 physician-investigators who completed surveys, thus, represent a significant proportion of the total estimated population, making this the most comprehensive study of this population to date. The mean age of MD-PhD SSH alumni was 55 years, with a standard deviation of 9.1. Respondents to this survey graduated between the mid 1950’s and 1999. Figure 1 shows the years in which MD-PhD SSH trainees received their respective doctoral degrees. The majority of respondents received their MDs in the 1980s and their PhDs in the 1990s and there was a general increase in the number of degree awarded per decade. Women are under-represented in the sample, making up 20% of the population (although this trend is changing, as will be discussed further in the Conclusion [1]).

As undergraduates, a slight majority (56%) of these trainees majored or minored in SSH disciplines as opposed to majoring in traditionally expected pre-medical basic science disciplines. Trainees completed their undergraduate degrees from a wide range of institutions, including public universities, private research universities, and small liberal arts colleges.

### Training paths

The training paths taken by MD-PhDs were remarkably diverse, including graduate degrees from multiple fields in SSH (see Table 1).

**Table 1:** Number of respondents by PhD discipline

PhD discipline	*n* = 55	Frequency (%)
Health services research	13	24
Anthropology (including all subfields)	12	22
History and Sociology of Science/Science Studies	11	20
Sociology	4	7.3
History, medical ethics, or public policy	3^a^	5.5^a^
Psychology or economics	2^a^	3.6^a^
Communications, philosophy, linguistics, or religion	1^a^	1.8^a^

^a^Refers to each individual discipline listed

In addition to choosing diverse PhD disciplines, SSH MD-PhD trainees reported taking many educational paths to obtain their dual degrees (see Table 2). The “traditional” path named in the table below reflects the pattern most common for the general MD-PhD population, beginning with the basic science years of medical school, followed by graduate training, and finishing with the clinical portion of medical school [1]. Fifty-five percent (30 out of 55) of SSH respondents trained through entirely “non integrated” mechanisms.

**Table 2:** Order of training

Stage of medical training when PhD was completed	*n* = 55	Frequency (%)
PhD completed prior to the initiation of medical school	4	7.3
PhD begun before medical school and completed during medical school training	3	5.5
Medical school started, then PhD started and completed in its entirety, then medical school completed (“Traditional Sequence”)	5	9.1
PhD completed simultaneously with medical school with studies in both fields every year	3	5.5
PhD completed after medical school training and before residency	2	3.6
PhD completed during residency	12	21.8
PhD completed after residency	26	47.3

Most SSH MD-PhDs (90%) in our sample completed clinical residency and choice in residency most commonly involved a primary care field (77.5%), followed by psychiatry (12.1%; see Table 3). In the context of the US, primary care fields include internal medicine (the inpatient and outpatient care of non-pregnant adults), pediatrics (the inpatient and outpatient care of children and youth), and family medicine (the inpatient and outpatient care of all demographic groups) [29].

**Table 3:** Number of respondents by medical specialty

Residency field	*n* = 58^a^	Frequency (%)
Internal medicine	19	32.7
Pediatrics	18	31.0
Psychiatry	7	12.1
Family medicine	5	8.6
Preventive medicine	3	5.2
Emergency medicine or surgery	2^b^	3.4^b^
OB/Gyn or pathology	1^b^	1.8^b^

^a^The total number (n) in this population is 58 because three people in our sample were board certified in two residency fields

^b^Refers to each individual specialty listed

### Years to completion of MD/PhD

The average number of years the physician-investigators took to finish their two degrees was 9 (SD 2.1). We found no statistically significant difference in the mean time to completion of degrees as correlated with age or the overall order of training of the two degrees.

**Fig. 2:**
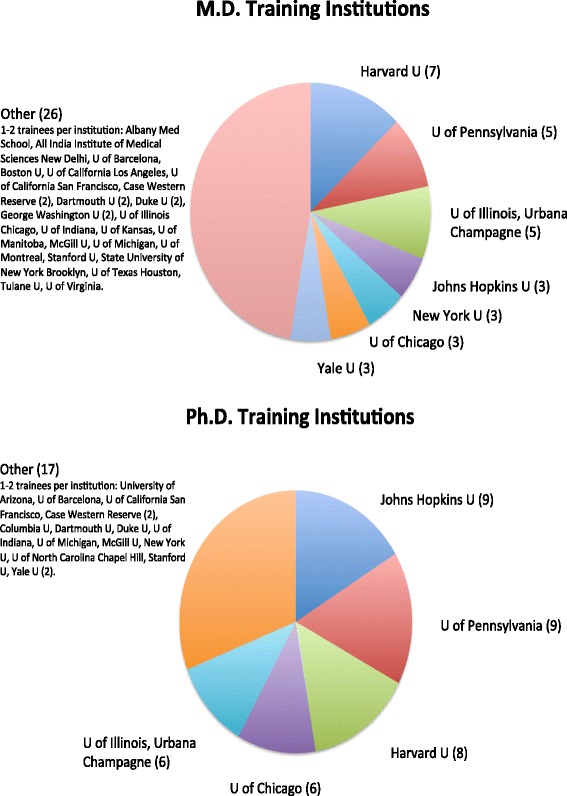
Institutions training MD/PhDs

Training for the MD occurred most often at Harvard University (7), the University of Pennsylvania (5), the University of Illinois Urbana Champaign (5), the Johns Hopkins University (3), New York University (3), the University of Chicago (3), and Yale University (3), which together represented the MD-granting institutions attended by 52.7% of respondents. Training for the PhD occurred most often at Johns Hopkins University (9), the University of Pennsylvania (9), Harvard University (8), the University of Chicago (6), and the University of Illinois Urbana Champaign (6), which together represented the PhD-granting institutions attended by 69% of respondents. As implied by the unequal representation of institutions, many trainees received their MDs and PhDs from different institutions, putting together their educations individually between different institutions without structured programmatic support (see Fig. 2 for full representation of training institutions).

### Post-education career

A large majority (88%) of our sample held faculty positions in academic institutions, nearly half (44%) of whom were mid-career or senior faculty (associate or full professor). More faculty members held primary appointments in social science or humanities departments (60%) than in clinical (22%) or combined departments (19%). Most of the non-faculty respondents (57%) held leadership positions in national or regional health organizations, both public and private, ranging from the Smithsonian Museum to the World Health Organization. Satisfaction with career path was not related to gender, age, completion of residency, or time to degree.

**Fig. 3:**
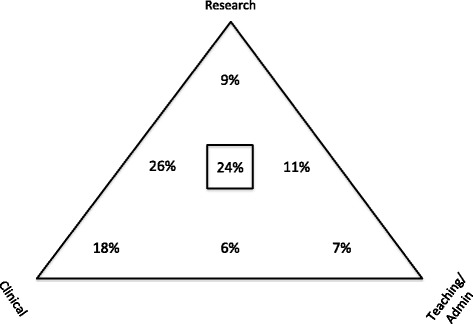
Proportion of time allocated by work activity

Respondents divided their time in myriad ways, with the majority of time in clinical work and research. Ninety-eight percent of respondents engaged in research. Only 4% of respondents worked in private practice. Figure 3 demonstrates the many permutations of career time allocation, graphically indicating the emphasis between the three poles of research, clinical work, and teaching/administration. As indicated in Fig. 3, 9% do primarily research, 7% primarily teaching/administration and 18% primarily clinical work. Of the remainder, 26% combine primarily clinical work and research, 11% research with teaching/administration, 6% clinical with teaching/administration, and 24% combine all three activities relatively equally.

### Perspectives on training

The vast majority (75%) responded that they were “optimistic about the ability of people to combine significant professional responsibilities in medicine and social sciences or humanities.” The most common explanations for this optimism were the experience of medicine and the SSH field enlightening one another (69%) and the experience of a supportive institution (23%). The most common reasons given for a lack of optimism were the pragmatic demands of clinical work (29%) and the lack of a supportive institutional environment (26%).

Optimism about integration was highly correlated with a generational effect. Among those who were optimistic, the average year of graduation (the year when the respondent finished the second degree) was 1991.6 (SD 6.2; *p* = .004) and the average year of graduation for those who were not optimistic was 1984.5 (SD 10.4; *p* = .004). Eighty nine percent of those who graduated after 1993 were optimistic about integration, while only 59% of respondents who graduated earlier than 1993 were optimistic (*p* = .03). Additionally, those who were optimistic about combining their degrees were more likely to have a clinical component to their career (OR 9.75, 95% CI 1.90-50.0). On the other hand, those who followed a sequence of training with no integration between the MD and the PhD in SSH were not likely to be optimistic about this combination (OR 0.24, 95% CI 0.06–0.92). Optimism about combining both degrees in a career was not related to gender, age, completion of residency, or the time it took to complete both degrees.

The results from the qualitative portion of the survey that explored encouraging and discouraging factors in pursuing joint training are provided in Table 4.

**Table 4:** Encouraging and discouraging factors in joint training process

Encouraging Factors	%	Discouraging factors	%
Interest/passion for work	50	Length of training	31
Supportive mentorship	32	Lack of role models	25
Institutional support	23	Financial difficulties	18
Benefit of an alternate perspective to pure medical training	13	Cultural gap between medicine and graduate field	18
Peer support	12		
No encouraging factors noted	10	No discouraging factors noted	25

In response to the “advantages and disadvantages of your educational pathway,” 40% recommend doing an MD first, 33% recommend doing the two programs simultaneously, 25% mention the benefit of clinical practice informing research, and 15% mention the benefit of both fields informing each other. Among those who recommended doing the MD first, 63% did the MD first. Of the 33% who recommended doing the two programs simultaneously, only one-third did it that way, and two-thirds obtained their MD and PhD degrees separately from each other.

When asked to provide recommendations for administrators and students engaged in building or pursuing these training paths, respondents emphasized several factors. Many (50%) stressed the need for programs to provide mentors. Respondents advocated both formal programmatic mentorship and networking by students themselves. Most (63%) also emphasized the responsibility and central role for scholars to advocate for the importance of social science and humanities to the practice of clinical medicine and the structure of the health care system.

Respondents provided concrete recommendations for how to establish or improve programs that provide joint training in SSH fields. Common recommendations included the following:

- Create flexibility to allow movement between medicine and SSH fields.
- Support and fund exploratory SSH research projects in medical school.
- Offer clinical involvement during PhD years.
- Establish dedicated program offices to help students with logistics, credits, scheduling, mentoring, and moral support.
- Provide leadership training programs, as success in SSH medical fields requires political astuteness, an understanding of policy and working within existing power dynamics.
- Involve students in leadership and administrative roles.
- Recognize faculty’s academic research, administrative work and teaching as well as clinical work as part of promotion.
- Find ways for mid-career MDs to return to PhD programs.
- Fund MD-PhD programs to prevent the stalling of progress caused by PhD students looking for funding.

### Research areas and directions

In response to the question, “what do you see as important areas or questions that need to be pursued by MD-PhDs in the social sciences or humanities,” respondents stressed the practical and theoretical importance of analyzing questions that appeal both to clinicians and to research colleagues as well as issues that affect patient care and health outcomes. For respondents, such questions fell into four major thematic areas.

First, respondents emphasized that SSH scholars should play a key role in training clinicians to be more sensitive and critical thinkers. A second common theme that emerged was the need to contextualize and provide a framework for understanding the culture of medicine itself. The third theme related to the utility of SSH research for understanding patients’ experiences, decisions and values. A final theme emphasized by many was the utility of SSH research to policy. As one respondent explained, “policy comes out of a complex social realm, so MD-PhDs in SSH fields are the best equipped of anyone to inform policy.”

## Discussion

Scholars have recently called for data on career outcomes for graduates of MD-PhD training programs as part of an overall project to evaluate the public funding of joint training [1–6]. To date, studies have addressed MD-PhD graduates in aggregate, ignoring potentially unique features of MD-PhDs trained in the social sciences and humanities [1, 8]. The present study seeks to fill this gap in the literature to understand this important subgroup.

Our data suggest that the vast majority of graduates of SSH MD-PhD programs have careers well aligned with the goals set forth by organizations that fund MD-PhDs, with some interesting differences from the general MD-PhD population. Like the general population of MD-PhD graduates described in Brass, et al. [1], the vast majority of our respondents (81%) reported careers matching what has been called “triple threat” – academic faculty positions combining clinical medicine, research, and teaching. Interestingly, many of the respondents in our survey also reported additional roles in administration and leadership of training programs or research centers – making their careers perhaps better described as “quadruple threat.” Another important difference is the diversity of educational backgrounds, undergraduate majors/minors, choices of graduate degrees, and research foci when compared with general MD-PhDs. A majority of our sample majored in non-traditional undergraduate degrees compared to 16% of undergraduates who majored in SSH and matriculated into medical school in 2013.^1^

In the 1960’s–1990’s, the relative dearth of institutionally-supported training programs forced many trainees to obtain each degree from separate institutions or to convince single institutions to allow combined degrees without a formal program. Although our survey did not directly address whether a graduate had trained as part of a formal joint MD-PhD program, we can obtain a clear sense for this in our population: Ninety one percent of graduates followed a sequence of degrees that deviates from the “traditional” order mandated by most formal joint programs; 34% of scholars studied at different institutions for each degree. Prior studies of the general MD-PhD population have focused only on graduates from structured programs and Brass et al. [1] estimate that the vast majority of MD-PhDs in the basic sciences study in structured programs, making this an important difference with our study population. The instability inherent in piecemeal training may contribute to the frustration expressed in responses from the earliest cohort in our sample. As more structured training programs include SSH, they may allow trainees to focus more on their education, improving satisfaction and decreasing time to completion.

The time to completion of the graduate degree in our sample is, on average, less than the general population of PhD students [30]. However, MD-PhDs in SSH took, on average, slightly longer than the overall MD-PhD sample described by Brass et al. (9 years versus 8 years [1]). This may be due to the increased time for the PhD in SSH fields overall or may be due to the relative lack of institutional support for such combined degrees, forcing many students to work or find other sources of funding during their studies. It may simply reflect the challenges of completing degrees outside of a structured combined degree program.

Other meaningful differences emerge when our survey population is compared with the overall MD-PhD population. Our respondents were more likely to have entered clinical fields in primary care (77.5%) and psychiatry (12.1%), rather than clinical sub-specialties, which are pursued by the majority of general MD-PhDs [1]. According to a recent *Academic Medicine* article on career paths of general MD-PhD trainees from MSTP programs, only 37% entered primary care fields compared to 43.6% of medical school graduates not in MSTP programs who participated in the 2004–2008 categorical match [2]. In contrast to this as mentioned above, primary care fields were the most common in our sample. Importantly, compared to MD-PhDs as a whole (16%), fewer (4%) SSH MD-PhDs worked in private practice [1]. Primary care fields are currently decreasing in popularity overall due to trends in the general population of MDs choosing specialties that fit “lifestyle choices” [31, 32]. Our respondents’ comments imply that they chose less specialized paths in part because they were looking for clinical settings that inspired socially relevant research questions, because they needed medical practices that allowed time for intensive field and archival research outside of the immediate clinical setting, and because these clinical areas supported a broad-minded curiosity for a diversity of situations and problems. These themes are evident in respondents’ opinions regarding important research questions, many of which were broad in nature, including understanding the culture of medicine, healthcare ethics, healthcare policy and medical education. Similar to the general MD-PhD population (88 to 95%) [1, 2, 5], most SSH MD-PhDs (90%) in our sample completed clinical residency.

A generational effect was found in this sample in terms of optimism regarding such combined training; optimism increased with later cohorts. Respondents’ qualitative answers implied that this was a result of an earlier cohort of trainees not having the benefit of mentorship from earlier trainees, not having formal funding or training programs in the 1950’s–1960’s and having had to create their own training piecemeal. Once MSTP sites and other programs began to support SSH MD-PhD students in the 1970s and 1980s, it appears that trainees have been able to complete their two degrees in a more integrated and supported fashion. The entry of this first cohort of SSH physician-investigators into the workforce involved building institutional structures and informal networks de novo that now support younger cohorts of trainees. In contrast to ambivalence found in the general population of MD-PhD trainees in Ahn et al.’s [33, 34] surveys, and in contrast to older generations, this younger cohort reports higher optimism about integrating their career. This may be due to benefiting from not only institutional support but also increasing relevant mentorship that was not available to earlier cohorts. As indicated by Andriole and Jeffe [8], formal institutional and funding support results in high rates of full-time faculty appointments within the general MD-PhD population. It would be reasonable to assume a similar relationship for MD-PhDs in SSH, though this is an empirical question for future research. Of note, trainees in joint SSH degree programs have recently formed a national organization, the Society for Humanities and Social Sciences and Medicine (SHSSM) that supports biennial conferences, research and professional collaborations [10].

This survey is the first to characterize this unique subset of physician-investigators with MD-PhD training in SSH. It points to many ways in which this population differs from the broader group of MD-PhDs and the central ways in which this subgroup meets the funding goals of MD-PhD training programs. In addition, this survey indicates that MD-PhDs in SSH meet more recent calls from such national bodies as the Institute of Medicine, the Department of Health and Human Services, and the American Academy of Medical Colleges for scholars who can understand increasingly complex connections between health, healthcare, society, social inequalities, and the globe [18, 20, 21]. Indeed, there is growing recognition of the importance of social structural influences on health and healthcare [15, 16, 35–37], which physician-investigators in SSH are uniquely qualified to address. For these diverse reasons, it is especially important now to support trainees in these areas.

This first survey of MD-PhDs in SSH has several limitations. We hope these limitations spur further inquiry. First, although the number of respondents in the survey may represent a significant proportion of the overall estimated population of SSH scholars in the timeframe surveyed, it was too small to power analysis of many factors that would help characterize the population. Moreover, the survey population of SSH graduates may contain an additional ascertainment bias, given that respondents were recruited through a combination of conferences, structured programs, and intensive internet searches. These searches may have under-selected graduates who are out of the academic or public eye. A study able to recruit the full population would be welcome, but given the lack of a national database of MD-PhD graduates in general, multi-pronged recruitment methods like ours are likely the best available. A more comprehensive study will hopefully be feasible in coming years, given that an increasing number of MD-PhDs in SSH are training in structured programs, and trainees are beginning to organize and track their progress via organizations such as APSA and SHSSM [10].

In addition, an increasingly large number of physician-investigators are currently graduating and being trained, and our study does not include these newer cohorts. The increase in trainees occurred over the last decade with the significant expansion of programs that offer structured combined SSH degree programs. This most recent set of trainees had not obtained their degrees by the time this survey of graduates was concluded. We hope this study will serve as necessary background and context for future research with this growing population to provide information about how the landscape of SSH MD-PhD training and careers is transforming and what these physician-investigators are contributing to health and healthcare.

## Conclusion

The data from our survey indicate that MD-PhDs in SSH manifest the goals of physician-investigator training in interesting and diverse ways. The vast majority integrates research, clinical work, and teaching in their career, matching the “triple threat” goal of public funding for combined physician-investigator training. Many also hold important administrative and leadership positions, including prominent figures such as Jim Yong Kim (President of the World Bank), Paul Farmer (Co-Founder of Partners in Health), and Camara Jones (Medical Officer at the Center for Disease Control and Prevention and President of the American Public Health Association). As such, MD-PhDs in SSH may be considered a “quadruple threat” when administration and leadership is added as the fourth valuable pole of their integrated careers. Given the growing awareness of the social structural factors producing health inequalities and problems in the responses of health systems in the US and around the world [15, 16, 18, 20, 21, 35–37], training physician-investigators with expertise in the social sciences and humanities must be a top priority. With this dual training, these clinician-scholars are uniquely prepared to respond to these critical problems in health, healthcare, and global health.

Different from the general MD-PhD population, most physician-investigators in SSH enter primary care or psychiatry instead of the medical subspecialties for their clinical training and fewer MD-PhDs in SSH enter private practice than the general MD-PhD population. Also different from the MD-PhDs in the basic biological, chemical and physical sciences, many physician-investigators in SSH have had to piece together their own training outside of formal programs, sometimes completing degrees at different institutions. However, perhaps because of the match between SSH MD-PhD training and the overall goals in public funding for physician-investigators, the number of institutions with structured combined programs in SSH has more than quintupled over the last decade.

For programs and program directors interested in supporting the SSH physician-investigator population, this survey provides potentially helpful insights that likely have similar implications inside and outside the US context. The primary areas respondents identified as key to supporting SSH MD-PhDs include the need for more role models or mentors upon whom young scholars can rely for guidance and career advice, the need for funding and institutional support, as well as the need for opportunities for funding for health-related SSH research after graduation, especially in early career.

In summary, physician-investigators in the social sciences and humanities meet the goals of public funding for MD-PhD training. They integrate research, clinical work, teaching as well as administration and leadership in order to address critical questions in health and healthcare in our contemporary society and globalizing world. The data from this project indicate that training for MD-PhDs in the social sciences and humanities should receive strong programmatic support and funding.

## Additional file

Additional file 1: Survey Instrument. (DOCX 105 kb)

## Abbreviations

MSTP: Medical scientist training program
NIH: National Institutes of Health
SSH: Social sciences and humanities

## Footnotes

1 The AAMC data at https://www.aamc.org/data/facts/applicantmatriculant/includes the following categories as possibilities for undergraduate majors: biological sciences, humanities, mathematics and statistics, other, physical sciences, social sciences, health sciences. If we include “humanities,” “social sciences,” and “other” from the 2013 AAMC data, the percentage of 2013 medical school matriculates receiving those undergraduate degrees in non-science majors would increase to 32%.
