# Supplementary material for: The first nationwide survey of MD-PhDs in the social sciences and humanities: training patterns and career choices
Source: BMC Med Educ. 2017 Mar 21;17:60. doi: 10.1186/s12909-017-0896-1 (PMC5361808; doi:10.1186/s12909-017-0896-1)
Supplement: Additional file 1: — Survey Instrument. (DOCX 105 kb) [file 12909_2017_896_MOESM1_ESM.docx]

SURVEY OF M.D./PH.D.’s IN THE SOCIAL SCIENCES AND HUMANITIES

Diane L. Gottheil, Ph.D. and Harold M. Swartz, M.D., Ph.D.

We are taking advantage of the occasion of the National Meeting of Social Science M.D./Ph.D. Students and Graduates to survey those of you who already hold both degrees, the "graduates," on your career paths and words of wisdom. We are hoping that this will provide useful information to add to the next edition of our book, The Education of Physician-Scholars: Preparing for Leadership in the Health Care System (Rockville, MD: Betz Publishers, Inc., 1993). The book sends a strong message about the need for and the shortage of physicians educated to conduct scholarly research in the social sciences and humanities and to provide leadership in education and policy arenas on the many issues in medicine and society germane to these disciplines. We hope you will be willing to take the time to respond to the questions that follow and to provide some data as a start toward understanding what needs to be done to encourage the development of programs and resources for M.D./Ph.D. students in the social sciences and humanities. **(Note: If you prefer to refer to your curriculum vita to answer some of the following items, please provide a copy when you return this questionnaire.)**

1. **Undergraduate Education:** Degree(s) and Year(s) Awarded: ________________

Major Field(s): ________________

Minor Field(s): ________________

Institution(s): ________________

2. **Graduate and Medical Education:**

1. Degrees and Years Awarded:

M.D. Awarded 19__ Institution: ________________

Ph.D. Awarded 19__ Institution: ________________

1. Sequence of Studies: Please describe the sequence of your M.D. and Ph.D. studies, include graduate medical education, clinical fellowships, or postgraduate research fellowships that preceded the award of the whichever degree came second. Be as detailed as possible and indicate any interruptions in formal program of studies (e.g. research and/or clinical and/or teaching position.)

Institution Discipline

Degree Yes__

Year 1: 19__ __________________________________________ Program? No__

Degree Yes__

Year 2: 19__ __________________________________________ Program? No__

Degree Yes__

Year 3: 19__ __________________________________________ Program? No__

Degree Yes__

Year 4: 19__ __________________________________________ Program? No__

Degree Yes__

Year 5: 19__ __________________________________________ Program? No__

Degree Yes__

Year 6: 19__ __________________________________________ Program? No__

Degree Yes__

Year 7: 19__ __________________________________________ Program? No__

Degree Yes__

Year 8: 19__ __________________________________________ Program? No__

Degree Yes__

Year 9: 19__ __________________________________________ Program? No__

Degree Yes__

Year 10: 19__ __________________________________________ Program? No__

Degree Yes__

Year 11: 19__ __________________________________________ Program? No__

Degree Yes__

Year 12: 19__ __________________________________________ Program? No__

Degree Yes__

Year 13: 19__ __________________________________________ Program? No__

Degree Yes__

Year 14: 19__ __________________________________________ Program? No__

Degree Yes__

Year 15: 19__ __________________________________________ Program? No__

(Please use back of this page for additional years, if needed.)

3. **Post-M.D./Ph.D. Training:**

Please describe residency, fellowship, or other formal training that followed the award of the M.D. and Ph.D. degrees. (This assumes that if such training came between the award of the t w o degrees it is noted in your response to question 2B, above.)

4. How satisfied are you with the sequence of studies that you followed? (Circle one:)

Very satisfied (1), Somewhat Satisfied (2), No Opinion (3), Somewhat Unsatisfied (4), Very Unsatisfied (5)

5. Please comment on the advantages of disadvantages of your educational pathway and any recommendations on sequence of studies you would have to those thinking of M.D./Ph.D. studies in the social sciences or humanities:

6. What were the factors that motivated you or provided encouragement in pursuing this degree combination?

7. What was discouraging?

8. What other recommendations would you have for students or program administrators?

9. What do you see as the important areas or questions that need to be pursued by M.D./Ph.D.s in the social sciences or humanities? (Please indicate whether this is your research area.)

10. In your current position, please estimate how you divide your time among the following responsibilities:

(circle one)

1. Clinical/Patient Care: __________% time per week/ month/ year;
2. Research: __________% time per week/ month/ year;
3. Teaching: __________% time per week/ month/ year;
4. Administration: __________% time per week/ month/ year;
5. Other: ________________ __________% time per week/ month/ year;

(specify)

1. Other: ________________ __________% time per week/ month/ year;

(specify)

11. Throughout your career, to what extent have you integrated your scholarly and leadership pursuits with the practice of medicine? (Circle one:)

Very Integrated (1), Somewhat Integrated (2), Not too Integrated (3), Not at all Integrated (4)

12. Please comment on how optimistic you are or are not about the ability of people to combine significant professional responsibilities in medicine and social sciences or humanities.

13. In what year were you born? ___________

14. Are you: A. Male ………..1

B. Female …….2

Your name (optional): __________________________________________________________________

Title: __________________________________________________________________

Institution: __________________________________________________________________

The data from this survey are confidential and only to be presented in an aggregate form. Please return this questionnaire to:

Diane L. Gottheil, Ph.D.,

Associate Director Medical Scholars Program

University of Illinois College of Medicine

at Urbana-Champaign

125 M 8 8 - 506 South Mathews (MC‐714)

Urbana,IL 61801

(Please do not hesitate to call Diane Gottheil if you have any questions concerning this survey: 217/333-8146.)
